# Age-Related Changes in Hepatic Lipid Metabolism and Abdominal Adipose Deposition in Yellow-Feathered Broilers Aged from 1 to 56 Days

**DOI:** 10.3390/ani13243860

**Published:** 2023-12-15

**Authors:** Ruixia Lan, Linlin Wei, Haibin Yu, Ping Jiang, Zhihui Zhao

**Affiliations:** Department of Animal Science and Technology, College of Coastal Agriculture Sciences, Guangdong Ocean University, Zhanjiang 524088, China; lanrx@gdou.edu.cn (R.L.); weill@stu.gdou.edu.cn (L.W.); yuhb@gdou.edu.cn (H.Y.); jiangp@gdou.edu.cn (P.J.)

**Keywords:** hepatic lipid metabolism, abdominal adipose deposition, age-related changes, yellow-feathered broilers

## Abstract

**Simple Summary:**

The aim of this study was to elucidate the age-related changes in hepatic lipid metabolism and abdominal adipose deposition in yellow-feathered broilers. Parameters such as body and abdominal adipose weight and lipid-metabolism-related gene expression in the liver and abdominal adipose tissue were evaluated. The results demonstrated that the body weight and absolute and relative weights of the liver increased with age-related changes. The triacylglycerol content peaked on day 14, while the total cholesterol content peaked on day 56. The adipocyte diameter and area peaked on day 56, the total DNA content peaked on day 7, the mRNA expression of hepatic *ChREBP*, *SREBP-1c*, *ACC*, *FAS*, *SCD1*, *CPT1*, *ApoB*, and *FABP1* peaked on day 7, *PPARα* on day 56, *LPL* on day 1, and *MTTP* on day 35. In abdominal adipose tissue, the mRNA expression of *PPARα*, *CPT1*, and *LPL* peaked on day 56, *PPARγ* on day 14, *C/EBPα* on day 42, and *C/EBPβ* on day 7. In addition, the age-related changes in the expression of hepatic lipogenesis- and lipolysis-related genes and abdominal adipose-deposition-related genes occurred during days 1 to 14 and during days 1 to 21, respectively. These results support the development of practical strategies to regulate hepatic lipid metabolism and reduce abdominal adipose deposition in yellow-feathered broilers.

**Abstract:**

The objective of this study was to evaluate the age-related changes in hepatic lipid metabolism, adipocyte hyperplasia, hypertrophy, and lipid metabolism in the abdominal adipose tissue of yellow-feathered broilers. Blood, liver, and abdominal adipose samples were collected on days 1, 7, 14, 21, 28, 35, 42, 49, and 56. Body, liver, and abdominal weight increased (*p* < 0.05) with age-related changes. The triacylglycerol content peaked on day 14, and total cholesterol content peaked on day 56. The adipocyte diameter and area peaked on day 56, and total DNA content peaked on day 7. The age-related changes in hepatic lipogenesis-related gene (*ChREBP*, *SREBP-1c*, *ACC*, *FAS*, *SCD1*) expression mainly occurred during days 1 to 21, hepatic lipolysis-related gene (*CPT1*, *LPL*, *ApoB*) expression mainly occurred during days 1 to 14, and abdominal adipose-deposition-related gene (*PPARα*, *CPT1*, *LPL*, *PPARγ*, *C/EBPβ*) expression occurred during days 1 to 14. These results demonstrated a dynamic pattern of hepatic lipid metabolism and abdominal adipose deposition in yellow-feathered broilers, which provides practical strategies to regulate hepatic lipid metabolism and reduce abdominal adipose deposition in yellow-feathered broilers.

## 1. Introduction

In recent decades, commercial broilers have been intensively selected for growth rate, meat production, and feed utilization, which are inevitably accompanied by excessive abdominal adipose deposition, increased mortality, and metabolic disorders [1,2]. Excessive abdominal adipose deposition is not desired by producers or consumers due to its detrimental effects on feed utilization, carcass characteristics, meat quality, economic concerns, and potential health issues of consumers [3,4,5]. A recent report stated that over 3 million tons of abdominal adipose tissue is produced in the poultry industry around the world every year, which directly leads to over USD 2.7 billion in economic loss [6]. In poultry, the liver is the main organ of de novo fatty acid synthesis, and abdominal adipose tissue serves as the main lipid storage site. Hepatic synthetic fatty acid is transported as lipoproteins via the bloodstream to abdominal adipose tissue for storage as triacylglycerol in lipid droplets in adipocytes [7]. Former studies demonstrated that several lipid-metabolism-regulating pathways influence and modulate abdominal adipose deposition, and hepatic lipid metabolism is the most important [8,9]. Thus, elucidation of the dynamic changes in hepatic lipid metabolism, abdominal adipose accumulation, and the underlying mechanisms is needed urgently and will provide insights into nutritional manipulation strategies to regulate lipid metabolism and decrease abdominal adipose deposition. Furthermore, the development of adipose tissue consists of an increase in adipocyte number (hyperplasia) and size (hypertrophy), increases with age, and is highly associated with body and abdominal adipose weight [10]. In other words, hepatic lipid metabolism as well as adipocyte hyperplasia and hypertrophy contribute to abdominal adipose deposition in broilers. The development of adipose tissue is regulated by various factors, including nutritional manipulation and hormonal and transcription factors [11,12,13,14]. In poultry, some experiments were conducted to evaluate lipid metabolism and abdominal adipose deposition in different broiler lines or weights [12,15,16]. However, reports on abdominal adipose development in yellow-feathered broilers are scarse. Feed constitutes over 60% of total broiler production costs; thus, maximizing the feed utilization efficiency is crucial from an economical perspective [17]. In addition, broilers are widely used as a model to study adipose tissue development as well as to mimic early-stage type 2 diabetes in humans [18,19,20]. Therefore, evaluating the age-related changes in abdominal adipose deposition and the underlying cellular and molecular mechanisms will broaden our understanding of adipose tissue development and hepatic lipid metabolism in yellow-feathered broilers. Meanwhile, these results may also support the development of practical strategies for using feed additives to regulate hepatic lipid metabolism and reduce abdominal adipose deposition in yellow-feathered broilers as well as support developments in biomedical research. We hypothesized that lipid metabolism changes with age, including hepatic lipid metabolism and abdominal adipose deposition. To verify our hypothesis, this study was conducted to investigate the age-related changes in hepatic lipid metabolism, adipocyte hyperplasia and hypertrophy, and lipid metabolism in the abdominal adipose tissue of yellow-feathered broilers.

## 2. Materials and Methods

### 2.1. The Experimental Design, Diets, and Management

A total of 240 one-day-old female, yellow-feathered broilers were randomly allocated to 6 replicates of 40 broilers each, and the yellow-feathered broilers were raised in ladder cages with 10 broilers each. The basal diet used is described in our former study and provided in the Supplementary Table S1 [21]. Management procedures were conducted according to feeding management regulations of yellow-feathered chickens (NY/T 1871-2010). The use of yellow-feathered broilers and experimental protocols were approved by the Animal Care and Use Committee of Guangdong Ocean University, China (SYXK-2018-0147).

### 2.2. Sample Collection

On days 1, 7, 14, 21, 28, 35, 42, 49, and 56, one broiler per replicate cage with a body weight close to the average was randomly selected and its body weight was checked; then, blood samples were collected from the wing vein and centrifuged at 3000× *g* for 15 min to collect serum samples and stored at −20 °C for later analysis. After that, these selected, yellow-feathered broilers were slaughtered by cervical dislocation, liver and abdominal adipose were quickly excised and weighted, about 1 g liver and abdominal adipose was rapidly collected into RNAase-free tubes, and then it was quickly frozen in liquid nitrogen and stored at −80 °C for later analysis. In addition, about 1 cm^3^ abdominal adipose was collected and fixed in 4% paraformaldehyde for 24 h at room temperature for later morphological analysis.

### 2.3. Triacylglycerol and Total Cholesterol Measurement

The pretreatment of the liver samples was according to the methods described in our former study [22]. The protein level was measured using the Bradford method (Cat. No. A045-4-2). Triacylglycerol (TG, Cat. No. A110-1-1) and total cholesterol (TC, Cat. No. A111-1-1) were detected using the corresponding kits (Nanjing Jiancheng Bioengineering Institute, Nanjing, China) following the instructions. The ratio of TG and TC to protein was calculated to express the TG and TC levels.

### 2.4. Morphological Analysis and Cellularity Determination

The morphological analysis of abdominal adipose tissue was according to the methods described by Guo et al. [23]. Stained sections were imaged with an Olympus BX51 microscope, and Image-Pro Plus 6.0 was used to determine the adipocyte area. For each section, we captured 6 images, and all adipocyte areas within the image field were measured; adipocyte areas larger than 20 μm^2^ were recorded, and we calculated the mean adipocyte diameter. 

### 2.5. Total DNA Content in Abdominal Adipose

Genomic DNA was extracted from approximately 100 mg of abdominal adipose wet tissue using a commercial DNA extraction kit (Cat. No. DP304-03, Tiangen Biotech Co., Ltd., Beijing, China). The DNA content was measured by using ultraviolet (UV) spectrophotometry, and it was demonstrated as μg/g of abdominal adipose wet tissue.

### 2.6. Gene Expression in Liver and Abdominal Adipose

Total RNA extraction was performed using a Trizol reagent kit (Accurate Biotechnology Co., Ltd., Changsha, China) and quantified with a UV spectrophotometer at 260 and 280 nm absorbance ratio. Meanwhile, the RNA integrity was verified via agarose gel electrophoresis. Then, a one-step gDNA removal kit (TransGen Biotech Co., Ltd., Beijing, China) was used to synthesize the cDNA, and a real-time quantitative polymerase chain reaction (RT-PCR) was performed as per our previously described methods [24]. The 2^−ΔΔCt^ method was used to calculate the relative gene expression, and β-actin was used as the internal reference gene [25]. The primer sequences used are provided in Supplementary Table S2.

### 2.7. Statistical Analysis

All data were analyzed with SAS 2003 (v. 9.1, SAS Institute Inc., Cary, NC, USA) using the GLM mixed procedure. Duncan’s multiple range test was used to analyze the differences among the age-related changes. *p* < 0.05 was considered as significantly different.

## 3. Results

### 3.1. Body, Liver, and Abdominal Adipose Weights

Age-related changes in body weight, and absolute and relative weights of liver and abdominal adipose in yellow-feathered broilers, are demonstrated in Table 1. Body weight increased from days 1 to 56 (*p* < 0.05). The liver weight remained stable from days 1 to 21, increased (*p* < 0.05) from days 21 to 42, decreased from days 42 to 49, and then increased (*p* < 0.05) from days 49 to 56. The abdominal adipose weight remained stable from days 1 to 14, increased from days 14 to 21, remained stable from days 21 to 28, and then increased (*p* < 0.05) from days 28 to 56. The relative weight of the liver remained stable from days 1 to 42, decreased (*p* < 0.05) from days 42 to 49, and remained stable until day 56. The relative weight of abdominal adipose remained stable from days 1 to 14, increased (*p* < 0.05) from days 14 to 21, decreased (*p* < 0.05) from days 21 to 28, remained stable from days 28 to 35, increased (*p* < 0.05) from days 35 to 42, remained stable from days 42 to 49, and then increased (*p* < 0.05) from days 49 to 56. 

### 3.2. Hepatic TG and TC Contents

**Figure 1:**
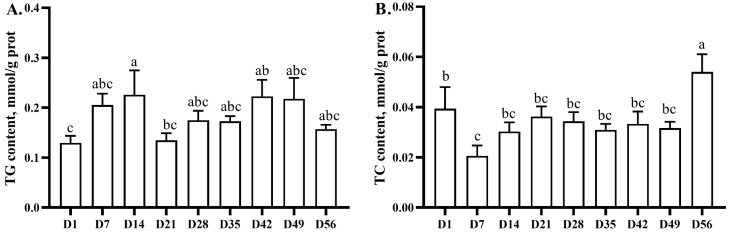
Age-related changes in hepatic triacylglycerol and total cholesterol contents of yellow-feathered broilers. TG, triacylglycerol; TC, total cholesterol; (**A**), TG content; (**B**), TC content. ^a–c^ Different lowercase letters indicate statistically significant differences (*p* < 0.05).

The hepatic TG content remained stable from days 1 to 14, decreased (*p* < 0.05) from days 14 to 21, and remained stable until day 56 (Figure 1). The TC content decreased from days 1 to 7, remained stable until day 49, and then increased (*p* < 0.05) from days 49 to 56.

### 3.3. Morphological and DNA Content Changes in Abdominal Adipose

**Figure 2:**
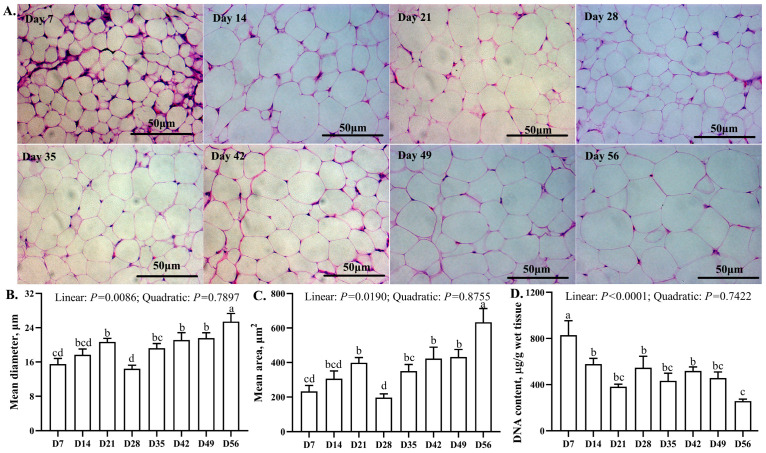
Age-related changes in the morphology, adipocyte size, and DNA content in abdominal adipose tissue of yellow-feathered broilers. (**A**) Morphology of abdominal adipose tissue; (**B**) mean diameter of abdominal adipose tissue; (**C**) mean area of abdominal adipose tissue; (**D**) DNA content of abdominal adipose tissue. ^a–d^ Different lowercase letters indicate statistically significant differences (*p* < 0.05).

The age-related changes in abdominal adipose tissue morphology are shown in Figure 2. The adipocyte diameter and area remained stable from days 7 to 21, decreased (*p* < 0.05) from days 21 to 28, increased from days 28 to 35, remained stable from days 35 to 49, and then increased (*p* < 0.05) from days 49 to 56. The biggest and smallest adipocyte diameter and area were observed on days 28 and 56, respectively. The total DNA content decreased (*p* < 0.05) from days 7 to 14 and remained stable from days 14 to 56. The highest and lowest DNA contents were observed on days 7 and 56, respectively. 

**Figure 3:**
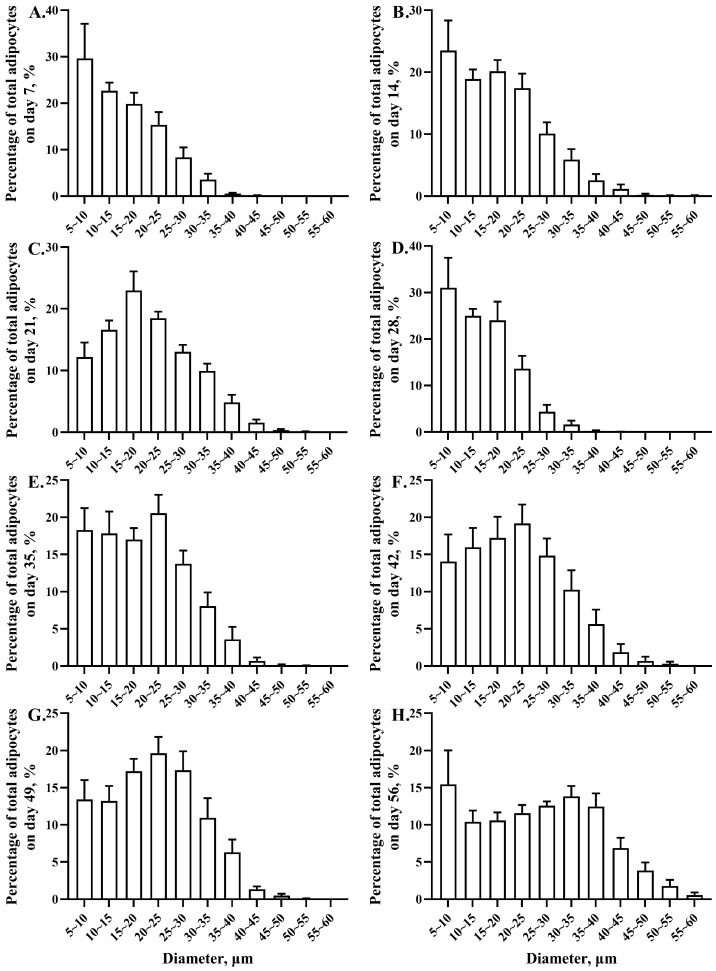
Age-related changes in adipocyte diameter distributions in abdominal adipose of yellow-feathered broilers. The adipocyte diameter distributions in abdominal adipose on days 7 (**A**), 14 (**B**), 21 (**C**), 28 (**D**), 35 (**E**), 42 (**F**), 49 (**G**), and 56 (**H**).

The adipocyte size distribution differed with age-related changes (Figure 3). On day 7, most (87.44%) of the adipocyte diameters ranged from 5 to 25 μm. The adipocyte diameters that ranged from 25 to 30 μm comprised 8.35% of the total number of adipocytes, the larger adipocytes (mean diameter range from 30 to 45 μm) comprised 4.21%, and no adipocytes greater than 45 μm were observed. On day 14, most (79.95%) of the adipocyte diameters ranged from 5 to 25 μm. The adipocyte diameters that ranged from 25 to 30 μm comprised 10.08% of the total number of adipocytes, the larger adipocytes (mean diameter range from 30 to 60 μm) comprised 9.97%, and there were adipocytes as large as 55 to 60 μm in diameter (0.09%). On day 21, most (70.23%) of the adipocyte diameters ranged from 5 to 25 μm. The adipocyte diameters that ranged from 25 to 35 μm comprised 22.97% of the total number of adipocytes, the larger adipocytes (mean diameter range from 35 to 55 μm) comprised 6.80%, and there were adipocytes as large as 50 to 55 μm in diameter (0.09%). On day 28, most (96.37%) of the adipocyte diameters ranged from 5 to 20 μm. The adipocyte diameters that ranged from 25 to 30 μm comprised 4.37% of the total number of adipocytes, the larger adipocytes (mean diameter range from 30 to 60 μm) comprised 1.96%, and there were adipocytes as large as 55 to 60 μm in diameter (0.03%). On day 35, most (73.71%) of the adipocyte diameters ranged from 5 to 25 μm. The adipocyte diameters that ranged from 25 to 30 μm comprised 13.76% of the total number of adipocytes, the larger adipocytes (mean diameter range from 30 to 55 μm) comprised 12.52%, and there were adipocytes as large as 50 to 55 μm in diameter (0.04%). On day 42, most (66.43%) of the adipocyte diameters ranged from 5 to 25 μm. The adipocyte diameters that ranged from 25 to 35 μm comprised 25.09% of the total number of adipocytes, the larger adipocytes (mean diameter range from 35 to 55 μm) comprised 8.49%, and there were adipocytes as large as 50 to 55 μm in diameter (0.30%). On day 49, most (63.52%) of the adipocyte diameters ranged from 5 to 25 μm. The adipocyte diameters that ranged from 25 to 35 μm comprised 28.29% of the total number of adipocytes, the larger adipocytes (mean diameter range from 35 to 55 μm) comprised 8.19%, and there were adipocytes as large as 50 to 55 μm in diameter (0.06%). On day 56, most (48.04%) of the adipocyte diameters ranged from 5 to 25 μm. The adipocyte diameters that ranged from 25 to 40 μm comprised 38.89% of the total number of adipocytes, the larger adipocytes (mean diameter range from 40 to 60 μm) comprised 13.07%, and there were adipocytes as large as 50 to 60 μm in diameter (2.33%).

### 3.4. Hepatic Lipid-Metabolism-Related Gene Expression

**Figure 4:**
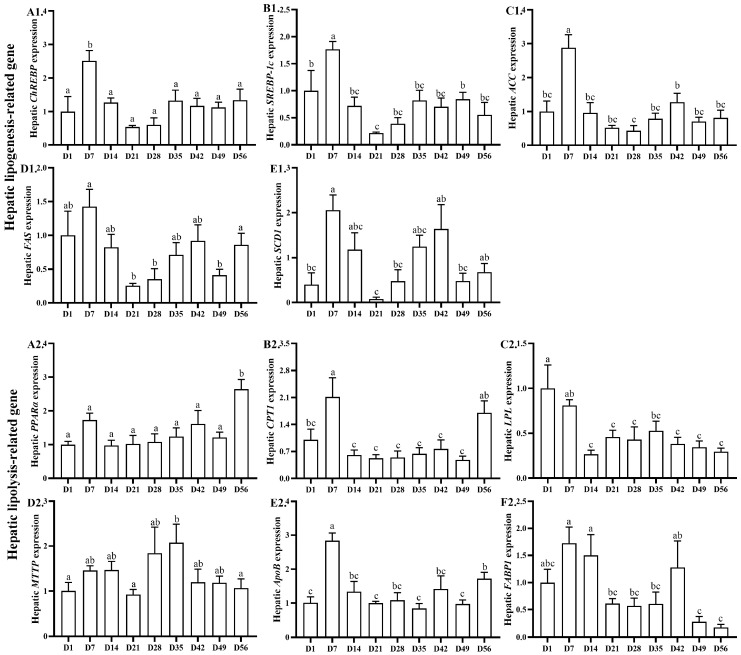
Age-related changes in hepatic lipogenesis- and lipolysis-related gene expression in yellow-feathered broilers. The relative mRNA expression levels of *ChREBP* (**A1**), *SREBP-1c* (**B1**), *ACC* (**C1**), *FAS* (**D1**), *SCD* (**E1**), *PPARα* (**A2**), *CPT1* (**B2**), *LPL* (**C2**), *MTTP* (**D2**), *ApoB* (**E2**), and *FABP* (**F2**). ^a–c^ Different lowercase letters indicate statistically significant differences (*p* < 0.05).

The age-related changes in hepatic lipid-metabolism-related gene expression are shown in Figure 4. The relative mRNA expression levels of carbohydrate responsive element binding protein (*ChREBP*), sterol regulatory element-binding protein-1c (*SREBP-1c*), and acetyl-coenzyme carboxylase (*ACC*) increased (*p* < 0.05) from days 1 to 7, decreased (*p* < 0.05) from days 7 to 14, and remained stable from days 14 to 56. The highest and lowest expression levels of *ChREBP* and *SREBP-1c* were observed on days 7 and 21, and *ACC* was observed on days 7 and 28, respectively. The relative mRNA expression level of fatty acid synthase (*FAS*) remained stable from days 1 to 49 and increased (*p* < 0.05) from days 49 to 56. The highest and lowest expression levels were observed on days 7 and 21, respectively. The relative mRNA expression level of stearoyl-CoA desaturase-1 (*SCD1*) increased (*p* < 0.05) from days 1 to 7 and remained stable from days 7 to 56. The highest and lowest expression levels were observed on days 7 and 21, respectively. The relative mRNA expression level of proliferator-activated regulator (*PPARα*) remained stable from days 1 to 49 and increased (*p* < 0.05) from days 49 to 56. The highest and lowest expression levels were observed on days 56 and 1, respectively. The relative mRNA expression levels of carnitine palmitoyltransferase-1 (*CPT1*) and apolipoprotein B (*ApoB*) increased (*p* < 0.05) from days 1 to 7, decreased (*p* < 0.05) from days 7 to 14, remained stable from days 14 to 49, and increased (*p* < 0.05) from days 49 to 56. The highest and lowest expression levels of *CPT1* were observed on days 7 and 49, and for *ApoB*, they were observed on days 7 and 35, respectively. The relative mRNA expression level of lipoprotein lipase (*LPL*) remained stable from days 1 to 7, decreased (*p* < 0.05) from days 7 to 14, and remained stable from days 14 to 56. The highest and lowest expression levels were observed on days 1 and 14, respectively. The relative mRNA expression level of microsomal triglyceride transfer protein (*MTTP*) remained stable from days 1 to 56, and the expression level on day 35 was higher (*p* < 0.05) than that on days 1, 21, and 56. The highest and lowest expression levels were observed on days 35 and 21, respectively. The relative mRNA expression level of fatty acid binding protein (*FABP1*) remained stable from days 1 to 14, decreased (*p* < 0.05) from days 14 to 21, remained stable from days 21 to 42, decreased (*p* < 0.05) from days 42 to 49, and remained stable from days 49 to 56. The highest and lowest expression levels were observed on days 7 and 56, respectively.

### 3.5. Lipid-Metabolism-Related Gene Expression in Abdominal Adipose

**Figure 5:**
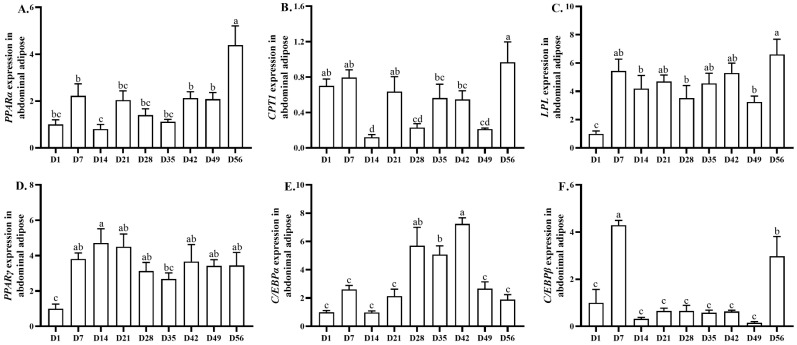
Age-related changes in lipolysis-, adipocyte-hyperplasia-, and hypertrophy-related gene expression in abdominal adipose tissue of yellow-feathered broilers. The relative mRNA expression of *PPARα* (**A**), *CPT1* (**B**), *LPL* (**C**), *PPARγ* (**D**), *C/EBPα* (**E**), and *C/EBPβ* (**F**). ^a–d^ Different lowercase letters indicate statistically significant differences (*p* < 0.05).

The age-related changes in lipid-metabolism-related gene expression in abdominal adipose tissue are shown in Figure 5. The relative mRNA expression level of *PPARα* remained stable from days 1 to 7, decreased (*p* < 0.05) from days 7 to 14, remained stable from days 14 to 49, and increased (*p* < 0.05) from days 49 to 56. The highest and lowest expression levels of *PPARα* were observed on days 56 and 14, respectively. The relative mRNA expression level of *CPT1* remained stable from days 1 to 7, decreased (*p* < 0.05) from days 7 to 14, increased (*p* < 0.05) from days 14 to 21, decreased (*p* < 0.05) from days 21 to 28, remained stable from days 28 to 49, and increased (*p* < 0.05) from days 49 to 56. The highest and lowest expression levels were observed on days 56 and 14, respectively. The relative mRNA expression level of *LPL* increased (*p* < 0.05) from days 1 to 7, remained stable from days 7 to 49, and increased (*p* < 0.05) from days 49 to 56. The highest and lowest expression levels were observed on days 56 and 1, respectively. The relative mRNA expression level of *PPARγ* increased (*p* < 0.05) from days 1 to 7 and remained stable from days 7 to 56. The highest and lowest expression levels were observed on days 14 and 1, respectively. The relative mRNA expression level of CCAAT/enhancer binding protein-*α* (*C/EBPα*) remained stable from days 1 to 21, increased (*p* < 0.05) from days 21 to 28, remained stable from days 28 to 35, increased (*p* < 0.05) from days 35 to 42, decreased (*p* < 0.05) from days 42 to 49, and remained stable from days 49 to 56. The highest and lowest expression levels were observed on days 42 and 1, respectively. The relative mRNA expression level of *C/EBPβ* increased (*p* < 0.05) from days 1 to 7, decreased (*p* < 0.05) from days 7 to 14, remained stable from days 14 to 49, and increased (*p* < 0.05) from days 49 to 56. The highest and lowest expression levels were observed on days 7 and 49, respectively.

## 4. Discussion

The age-related changes in abdominal adipose deposition can be explained in several ways, such as the changes in body weight, lipolysis, and TG metabolism [9]. In yellow-feathered broilers, the liver is the predominant organ for de novo fatty acid synthesis, and abdominal adipose tissue is specialized for fat storage [22]. In other words, abdominal adipose deposition is associated with hepatic fatty acid synthesis and the export of TG [9,26]. Age-related changes in abdominal adipose deposition and hepatic lipid metabolism have been described in animal models [27,28]. Guo et al. [23] also reported the age-related changes in body and abdominal adipose weight. Consistent with these results, we found age-related changes in the body and abdominal adipose weights. The liver in new-hatched broilers is lipid-rich, and the hepatic TG and TC contents increased to peaks at around the hatched day [26,29]. After hatching, broilers begin to consume feed, and hepatic lipid accumulation declined quickly during the first week post-hatching, while the lipid metabolism changed from depending on yolk to hepatic de novo lipogenesis and/or diet [30,31]. In this study, the hepatic TC content decreased from days 1 to 7, while the TG content remained stable, suggesting that hepatic TC was the main energy source from days 1 to 7, while hepatic de novo lipogenesis was limited. Consistent with our results, Liu et al. [26] reported that the hepatic TC content decreased from days 1 to 7.

There are age-related changes in abdominal adipose biology, including changes in absolute and relative weight, cellular composition, and abundance [32,33]. Abdominal adipose deposition is a result of hyperplasia and hypertrophy of adipose cells, which are associated with body and abdominal adipose weight [11,34]. To quantify the age-related changes in the size and number of adipocytes is vital in characterizing the phenotype of abdominal adipose deposition. In this study, we found that the abdominal adipose tissue weight changes coincided with adipocyte size and DNA content. Therefore, we speculated that adipocyte hypertrophy growth was the dominant manner of change in abdominal adipose. In this study, the total adipocytes were counted, and we subsequently calculated the frequency as a percentage of total adipocytes. Guo et al. [23] reported that adipose cell hyperplasia occurred from days 3 to 49, and adipose cell hypertrophic growth occurred from days 1 to 35. After that, hypertrophic growth was the predominant factor responsible for abdominal adipose deposition [10]. During abdominal adipose development, hyperplasia and preadipocyte differentiation are observed, and the increase in preadipocyte amount provides a foundation for differentiation to mature adipocytes and lipid accumulation. As each adipocyte contains the same DNA mass, total DNA mass in abdominal adipose is directly proportional to the number of adipocytes, which reflects hyperplastic growth [23,35]. A study in Leghorn chickens and broilers illustrated that there was an increase in total DNA mass of neck and leg adipose tissue during incubation from days 12 to 14, and it plateaued from incubation day 18 to post-hatching day 1 [35]. In this study, there was a decrease in total DNA mass from days 7 to 14, and that then remained stable until day 56, while unilocular mature adipocytes were notable on day 7, suggesting that the differentiation of preadipocytes finished during the incubation phase [35], and thus, that abdominal adipose deposition may not depend on hyperplastic growth in yellow-feathered broilers. Hypertrophic growth accompanies lipid deposition [36,37], and in this study, continuous increases in adipocyte diameter and area were observed from days 7 to 21 and days 28 to 56, suggesting that hypertrophic growth was the main contributor to abdominal adipose deposition [35,36]. Nevertheless, the abdominal adipose weight, adipocyte diameter, and area decreased from days 21 to 28. Abdominal adipose tissue is the main site for TG storage, and so the TG content reflected the TG storage capacity in abdominal adipose, as well as its weight [38]. The lower TG content, as well as the downregulated hepatic lipogenesis-related gene expression on days 21 and 28, may partly explain the decreasing abdominal adipose weight, adipocyte diameter, and area. 

In broilers, the liver is the main organ for de novo fatty acid synthesis, and the abdominal adipose serves as the main lipid storage site. Thus, abdominal adipose deposition depends on hepatic fatty acid synthesis and the utilization of serum TG by adipocytes [39,40]. To elucidate the molecular mechanism of lipid metabolism and abdominal adipose deposition, we further evaluated hepatic lipogenesis- and lipolysis-related gene expression. In this study, the hepatic *ACC* mRNA expression level increased from days 1 to 7, decreased from days 7 to 14, and remained stable from days 14 to 56, and the *SCD1* mRNA expression level increased from days 1 to 7 and then remained stable from days 7 to 56. However, previous studies reported that hepatic *ACC* and *FAS* mRNA expression levels increased to their peaks before hatching [26]. Meanwhile, the mRNA expression levels of hepatic *ChREBP* and *SREBP-1c* also increased from days 1 to 7, decreased from days 7 to 14, and remained stable from days 14 to 56, suggesting that *ChREBP* and *SREBP-1c* promoted hepatic lipogenesis at around day 14 in yellow-feathered broilers. 

Apart from hepatic lipogenesis, abdominal adipose deposition is also associated with transportation, lipid degradation, and fatty acid oxidation [8,41]. The synthesized hepatic fatty acids are incorporated into TG and secreted as very-low-density lipoprotein (VLDL). This illustrates how *MTTP* is responsible for the assembly and secretion of VLDL, as well as the incorporation of TG into *ApoB* [42]. *LPL* plays a vital role in lipid degradation, which catalyzes circulating TG hydrolysis from VLDL [43]. *LPL* is a tissue-specific enzyme, which is mainly expressed in adipose tissue and the liver of young animals [44]. In this study, we found that *LPL* was highly expressed in the liver of young, yellow-feathered broilers, and the hepatic *LPL* mRNA expression level decreased from days 7 to 14. Similar results were also reported in domestic pigeon squabs [45]. *PPARα* and *CPT1* play vital roles in lipolysis, which stimulates fatty acid oxidation [6]. In this study, the mRNA expression level of hepatic *PPARα* increased from days 49 to 56, while the mRNA expression level of *CPT1* increased from days 1 to 7 and days 49 to 56, but decreased from days 7 to 14. The hepatic *CPT1* mRNA expression level increased from days 1 to 7, suggesting that hepatic fatty acid oxidation was an important way to supply energy.

We also evaluated lipolysis- and preadipocyte-proliferation-, differentiation-, and maturation-related gene expression in abdominal adipose tissue. *PPARγ*, *C/EBPα*, and *C/EBPβ* play vital roles in adipocyte proliferation, differentiation and maturation [46,47]. In this study, the mRNA expression level of *PPARγ* increased from days 1 to 7, while the mRNA expression level of *C/EBPα* increased from days 35 to 42, but decreased from days 42 to 49, and the mRNA expression level of *C/EBPβ* increased from days 1 to 7 and days 49 to 56, but decreased from days 7 to 14. These results suggested that age-related changes in the mRNA expression levels of *PPARγ*, *C/EBPα*, and *C/EBPβ* were associated with abdominal adipose tissue weight. Furthermore, the mRNA expression level of *PPARα* decreased from days 7 to 14, but increased from days 49 to 56, while the mRNA expression level of *CPT1* decreased from days 7 to 14 and days 21 to 28, but increased from days 49 to 56. Meanwhile, the mRNA expression level of *LPL* increased from days 1 to 7 and days 49 to 56. The upregulated mRNA expression levels of *LPL* and *C/EBPβ* may partly explain the rapidly increasing abdominal adipose tissue weight during days 49 to 56. Although age-related changes in abdominal adipose-deposition-related gene expression were observed in this study, further research must be conducted to explain the underlying mechanisms.

## 5. Conclusions

In conclusion, this study has demonstrated age-related changes in hepatic lipid metabolism and abdominal adipose deposition in yellow-feathered broilers aged from 1 to 56 days. Our results have indicated that with age-related changes, hepatic lipid-metabolism-related genes illustrate different expression pattens. In particular, the expression of hepatic lipogenesis-related genes, including *ChREBP*, *SREBP-1c*, *ACC*, *FAS*, and *SCD1*, showed significant changes during days 1 to 21, and showed a “rise, decline, rise” trend. The hepatic lipolysis-related genes, including *CPT1*, *LPL*, and *ApoB*, showed significant changes during days 1 to 14, the mRNA expression levels of *CPT1* and *ApoB* showed a “rise, decline, rise” trend, and the mRNA expression level of *LPL* showed a decline trend. The mRNA expression levels of abdominal adipose-deposition-related genes, including *PPARα*, *CPT1*, *LPL*, *PPARγ*, and *C/EBPβ*, showed significant changes during days 1 to 14. The mRNA expression levels of *PPARα* and *C/EBPβ* showed a “rise, decline, rise” trend, the mRNA expression levels of *PPARγ* and *LPL* showed a rise trend, and the mRNA expression level of *CPT1* showed a decline trend. These results should support the development of practical strategies for regulating hepatic lipid metabolism and reducing abdominal adipose deposition.

## Figures and Tables

**Table 1:** Age-related changes in body weight and absolute and relative weights of liver and abdominal adipose in yellow-feathered broilers.

Item	Absolute Weight, g			Relative Weight, g/kg	
	Body Weight	Liver	Abdominal Adipose	Liver	Abdominal Adipose
Day 1	41.80 ± 5.11 ^a^	1.82 ± 0.23 ^g^	0.19 ± 0.05 ^f^	43.53 ± 2.99 ^a^	4.51 ± 0.86 ^g^
Day 7	111.99 ± 7.49 ^b^	4.38 ± 0.31 ^fg^	0.69 ± 0.13 ^f^	39.30 ± 4.57 ^ab^	6.23 ± 1.32 ^fg^
Day 14	181.24 ± 7.38 ^c^	6.21 ± 0.46 ^ef^	1.25 ± 0.13 ^f^	34.31 ± 2.77 ^bc^	6.91 ± 0.76 ^ef^
Day 21	305.32 ± 12.96 ^d^	8.85 ± 1.28 ^e^	3.78 ± 0.52 ^e^	28.93 ± 3.53 ^cd^	12.41 ± 1.92 ^bc^
Day 28	408.24 ± 30.25 ^e^	11.93 ± 3.39 ^d^	3.61 ± 0.40 ^e^	29.24 ± 8.14 ^cd^	8.83 ± 0.72 ^de^
Day 35	689.39 ± 5.47 ^f^	21.43 ± 3.08 ^c^	7.22 ± 0.89 ^d^	31.07 ± 4.31 ^c^	10.47 ± 1.28 ^cd^
Day 42	782.68 ± 16.49 ^g^	25.85 ± 4.40 ^b^	9.83 ± 2.62 ^c^	33.00 ± 5.33 ^c^	12.57 ± 3.37 ^b^
Day 49	922.90 ± 27.02 ^h^	22.64 ± 2.67 ^c^	12.51 ± 0.78 ^b^	24.51 ± 2.65 ^de^	13.58 ± 1.07 ^b^
Day 56	1415.72 ± 22.43 ^i^	32.40 ± 2.56 ^a^	37.69 ± 3.05 ^a^	22.89 ± 1.75 ^e^	26.62 ± 2.12 ^a^

^a–i^ Different lowercase letters in same column indicate statistically significant differences (*p* < 0.05).

## Data Availability

The data are available on request from the corresponding author.

## Supplementary Materials

The following supporting information can be downloaded at: https://www.mdpi.com/article/10.3390/ani13243860/s1, Table S1: Basal diet composition; Table S2: Primer sequences for real-time PCR.
