# Supplementary material for: Age-Related Changes in Hepatic Lipid Metabolism and Abdominal Adipose Deposition in Yellow-Feathered Broilers Aged from 1 to 56 Days
Source: Animals (Basel). 2023 Dec 15;13(24):3860. doi: 10.3390/ani13243860 (PMC10740587; doi:10.3390/ani13243860)
Supplement: Supplementary file 1 [file animals-13-03860-s001.zip › animals-2751137-supplementary.pdf]

animals

MDPI

Table S1. Basal diet composition (as-fed basis)

Items	Day 1 to 28	Day 29 to 56
Ingredients, %		
Corn	61.50	65.10
Soybean	28.50	23.00
Soybean oil	1.70	3.50
Calcium hydrogen phos-phate	1.93	1.65
Shell power	2.22	2.67
Salt	0.30	0.30
Methionine	0.17	0.12
Lysine	0.16	0.15
Zeolite powder	1.52	1.51
Vitamin premix[1]	1.00	1.00
Mineral premix[2]	1.00	1.00
Nutrient level		
Metabolic energy, MJ/kg	2.91	3.06
Crude protein, %	21.00	19.00
Crude fat, %	2.94	3.39
Calcium, %	1.00	0.90
Available phosphorous, %	0.45	0.41
Methionine, %	0.45	0.36
Lysine, %	1.13	0.98

[1] Provided per kilogram of complete diet: 12,8000IU Vitamin A, 1,600IU Vitamin D_3, 60IU Vitamin E, 1.6mg Vitamin K_3, 0.12mg Biotin, 50mg Choline, 1.2mg Folic acid, 32mg Nicotinic acid, 16mg Pantothenic acid, 4.8mg Riboflavin, 2.4mg Thiamine (VB_1), 3.2mg Vitamin B_6, and 0.03 mg Vitamin B_{12}.

[2] Provided per kilogram of diet: Mg, 79mg as manganese oxide; Zn, 60mg as zinc oxide; Cu 100mg as copper sulfate; Fe, 120mg as iron sulfate; I, 0.96mg as potassium iodine; Co, 0.16mg as cobalt sulfate and Se, 0.24mg as sodium selenite.

Table S2. Primers for real-time PCR

Gene[1]	Accession NO.	Primer sequence (5′ to 3′)	Product size, bp
β-actin	L08165	F: ATCCGGACCCTCCATTGTC<br>R: AGCCATGCCAATCTCGTCTT	113
ChREBP	EU152408	F: ATTGACCCGACCCTGACG<br>R: CATACTGGATGTACCACGCTCT	160
SREBP-1c	XM_015294109	F: GCCCTCTGTGCCTTTGTCTTC<br>R: ACTCAGCCATGATGCTTCTTC	130
FAS	J03860	F: TTTGGTGGTTCGAGGTGGTA<br>R: CAAAGGTTGTATTTCGGGAGC	215
ACC	J03541	F: GCTTCCCATTTGCCGTCCTA<br>R: GCCATTCTCACCACCTGATTACTG	185
SCD1	NM_204890	F: GTTTCCACAACTACCACCATACATT<br>R: CCATCTCCAGTCCGCATTTT	175
PPARα	AF163809	F: TTTAACGGA GTTCCA ATCGC<br>R: AACCCTTACAACCTTCACAAGC	224
MTTP	NM_001109784	F: GCAGATGGACAGAGTTGGCT<br>R: ACACCAAAAGTGCAAGGTGC	224
LPL	NM_205282	F: CCGATCCCGAAGCTGAGATG<br>R: ACATTCCTGTCACCGTCCAC	186
CPT1	AY675193	F: TAGAGGGCGTGGACCAATAA<br>R: TGGGATGCGGGAGGTATT	229
ApoB	NM_001044633	F: ATCAGCCAAGCAGAGCTTCC<br>R: GCCGAGCAGTGATACCATGA	122
FABP1	HQ640427.1	F: GAGCTCCAGTCCCATGAAAA<br>R: TCAGCAGCTCCATCTCACAC	120
PPARγ	NM_001001460	F: CACTGCAGGAACAGAACAAAGAA<br>R: TCCACAGAGCGAAACTGACATC	120
C/EBPα	NM_001031459	F: CGCGGCAAATCCAAAAAG<br>R: GGCGCACGCGGTACTC	120
C/EBPβ	NM_205253	F: GCCGCCCGCCTTTAAA<br>R: CCAAACAGTCCGCCTCGTAA	120

[1] *ChREBP*, carbohydrate responsive element binding protein, *SREBP-1c*, sterol regulatory element-binding protein 1c; *FAS*, fatty acid synthase; *ACC*, acetyl-coenzyme carboxylase; *SCD*, stearoyl-CoA desaturase; *PPAR*, proliferator-activated regulator; *MTTP*, microsomal triglyceride transfer protein; *LPL*, lipoprotein lipase; *CPT*, carnitine palmitoyl transferase; *ApoB*, Apolipoprotein B; *FABP*, fatty acid binding protein; *C/EBP*, CCAAT/enhancer binding protein.
